# Incidence and prevalence of primary biliary cholangitis in the Netherlands – A nationwide cohort study

**DOI:** 10.1016/j.jhepr.2024.101132

**Published:** 2024-06-06

**Authors:** Rozanne C. de Veer, Maria C.B. van Hooff, Ellen Werner, Ulrich Beuers, Joost P.H. Drenth, Frans J.C. Cuperus, Bart van Hoek, Bart J. Veldt, Michael Klemt-Kropp, Suzanne van Meer, Robert C. Verdonk, Hajo J. Flink, Jan Maarten Vrolijk, Tom J.G. Gevers, Cyriel Y. Ponsioen, Martijn J. ter Borg, Khalida Soufidi, Femke Boersma, Hendrik J.M. de Jonge, Frank H.J. Wolfhagen, L.C. Baak, Susanne L. Onderwater, Jeroen D. van Bergeijk, Paul G. van Putten, Gijs J. de Bruin, Rob P.R. Adang, Maria N. Aparicio-Pages, Wink de Boer, Frank ter Borg, Hanneke van Soest, Harry L.A. Janssen, Bettina E. Hansen, Nicole S. Erler, Adriaan J. van der Meer, the Dutch PBC Study Group, Sunje Abraham, Rob P.R. Adang, Huseyin Aktas, Yasser A. Alderlieste, Maria N. Aparicio-Pages, L. (Bert) C. Baak, Martine A.M.C. Baven-Pronk, A. (Sander) van der Beek, Frank C. Bekkering, Jeroen D. van Bergeijk, Ulrich Beuers, Menno Beukema, Wink de Boer, Femke Boersma, Kirsten Boonstra, Frank ter Borg, Martijn J. ter Borg, Pieter C.J. ter Borg, Gijs J. de Bruin, Paul J. Bus, Djuna L. Cahen, Marcel Cazemier, Frans J.C. Cuperus, Lisette J.H. van Dam, Maaike J. Denters, Joost P.H. Drenth, Ludger S.M. Epping, Nicole S. Erler, Hajo J. Flink, Philip W. Friederich, Nicole F.M. van Gerven, Tom J.G. Gevers, Bettina E. Hansen, Sven J. van den Hazel, Bart van Hoek, Maria C. van Hooff, Daphne M. Hotho, Harry L.A. Janssen, Hendrik J.M. de Jonge, Matthias C. Jurgens, J.(Netty) van Kemenade, Marjo J. Kerbert-Dreteler, Michael Klemt-Kropp, Ingrid C.A.W. Konings, Sander de Kort, Edith M.M. Kuiper, Johan P.H. Kuyvenhoven, Adriaan J. van der Meer, Suzanne van Meer, Susanne L. Onderwater, Leendert H. Oterdoom, Cyriel Y. Ponsioen, Paul G. van Putten, Janne E. van Rooij, Robert Roomer, Johannes Schmidt-Böhmer, Stephan Schmittgens, Tim C.M.A. Schreuder, Jerome Sint Nicolaas, Hanneke van Soest, Khalida Soufidi, Stephan H.C. van Stiphout, Hans H.K. Thio, Merel M. Tielemans, Sigrid Vandebosch, Rozanne C. de Veer, Bart J. Veldt, Robert C. Verdonk, J. Marleen de Vree, Elsemieke de Vries, Anne Vrieze, Jan Maarten Vrolijk, Laurens A. van der Waaij, Ellen Werner, Ulrike de Wit, Frank H.J. Wolfhagen

**Affiliations:** 1 Department of Gastroenterology and Hepatology, Erasmus University Medical Center, Rotterdam, the Netherlands; 2 Department of Gastroenterology and Hepatology, Amsterdam University Medical Center, Amsterdam, the Netherlands; 3 Department of Gastroenterology and Hepatology, Radboud University Medical Center, Nijmegen, the Netherlands; 4 Department of Gastroenterology and Hepatology, University Medical Center Groningen, Groningen, the Netherlands; 5 Department of Gastroenterology and Hepatology, Leiden University Medical Center, Leiden, the Netherlands; 6 Department of Gastroenterology and Hepatology Reinier de Graaf Gasthuis, Delft, the Netherlands; 7 Department of Gastroenterology and Hepatology, Noordwest Ziekenhuisgroep, Alkmaar, the Netherlands; 8 Department of Gastroenterology and Hepatology, University Medical Center Utrecht, Utrecht, the Netherlands; 9 Department of Gastroenterology and Hepatology, St. Antonius Hospital, Nieuwegein, the Netherlands; 10 Department of Gastroenterology and Hepatology, Catharina Hospital, Eindhoven, the Netherlands; 11 Department of Gastroenterology and Hepatology, Rijnstate, Arnhem, the Netherlands; 12 Department of Gastroenterology and Hepatology, Maastricht University Medical Center, Maastricht, the Netherlands; 13 Department of Gastroenterology and Hepatology, Maxima Medical Center, Eindhoven, the Netherlands; 14 Department of Gastroenterology and Hepatology, Zuyderland Medical Center, Heerlen, the Netherlands; 15 Department of Gastroenterology and Hepatology, Gelre Hospitals, Apeldoorn-Zutphen, the Netherlands; 16 Department of Gastroenterology and Hepatology, Jeroen Bosch Hospital, Den Bosch, the Netherlands; 17 Department of Gastroenterology and Hepatology, Albert Schweitzer Hospital, Dordrecht, the Netherlands; 18 Department of Gastroenterology and Hepatology, Onze Lieve Vrouwe Gasthuis, Amsterdam, the Netherlands; 19 Department of Gastroenterology and Hepatology, Diakonessenhuis, Utrecht, the Netherlands; 20 Department of Gastroenterology and Hepatology, Hospital De Gelderse Vallei, Ede, the Netherlands; 21 Department of Gastroenterology and Hepatology, Medical Center Leeuwarden, Leeuwarden, the Netherlands; 22 Department of Gastroenterology and Hepatology, Tergooi Hospital, Hilversum-Blaricum, the Netherlands; 23 Department of Gastroenterology and Hepatology, VieCuri, Venlo, the Netherlands; 24 Department of Gastroenterology and Hepatology, Canisius/Wilhemina Hospital, Nijmegen, the Netherlands; 25 Department of Gastroenterology and Hepatology, Bernhoven, Uden, the Netherlands; 26 Department of Gastroenterology and Hepatology, Deventer Hospital, Deventer, the Netherlands; 27 Department of Gastroenterology and Hepatology, Medical Center Haaglanden, Den Haag, the Netherlands; 28 Department of Gastroenterology and Hepatology, Toronto Centre for Liver Disease, University of Toronto, Ontario, Canada; 29 Department of Biostatistics, Erasmus University Medical Center, Rotterdam, the Netherlands; 30 Department of Epidemiology, Erasmus University Medical Center, Rotterdam, the Netherlands

**Keywords:** Primary biliary cholangitis, Incidence, Prevalence

## Abstract

**Background & Aims:**

Although primary biliary cholangitis (PBC) is considered a rare disorder, accurate determination of its incidence and prevalence remains challenging due to limited comprehensive population-based registries. We aimed to assess the incidence and prevalence of PBC in the Netherlands over time through the nationwide Dutch PBC Cohort Study (DPCS).

**Methods:**

DPCS retrospectively included every identifiable patient with PBC in the Netherlands from 1990 onwards in all 71 Dutch hospitals. Incidence and prevalence were assessed between 2008-2018 by Poisson regression between sex and age groups over time.

**Results:**

On the 1^st^ of January 2008, there were 1,458 patients with PBC in the Netherlands. Between 2008-2018, 2,187 individuals were newly diagnosed, 46 were transplanted and 468 died. The yearly incidence of PBC in 2008 was 1.38, increasing to 1.74 per 100,000 persons in 2018. When compared to those aged <45 years, females aged 45-64 years (adjusted incidence rate ratio 4.21, 95% CI 3.76-4.71, *p* <0.001) and males ≥65 years (adjusted incidence rate ratio 14.41, 95% CI 9.62-21.60, *p* <0.001) were at the highest risk of being diagnosed with PBC. The male-to-female ratio of patients newly diagnosed with PBC during the study period was 1:14 in those <45 years, 1:10 in patients aged 45-64 years, and 1:4 in those ≥65 years. Point prevalence increased from 11.9 in 2008 to 21.5 per 100,000 persons in 2018. Average annual percent change in this time period was 5.94% (95% CI 5.77-6.15, *p* <0.05), and was the highest among the population aged ≥65 years (5.69%, 95% CI 5.32-6.36, *p* <0.001).

**Conclusions:**

In this nationwide cohort study, we observed an increase in both the incidence and prevalence of PBC in the Netherlands over the past decade, with marked age and sex differences.

**Impact and implications:**

This nationwide Dutch primary biliary cholangitis (PBC) Cohort Study, including all hospitals in the Netherlands, showed that the incidence and prevalence of PBC have increased over the last decade. The age-dependent PBC incidence rate differed for males (highest risk ≥65 years) and females (highest risk between 45 and 65 years), which may be related to a difference in the timing of exposure to environmental triggers of PBC. The largest increase in PBC prevalence over time was observed in the population aged ≥65 years, which may have implications for the use of second-line therapies. These results therefore indicate that further studies are needed to elaborate on the advantages and disadvantages of add-on therapies in the elderly population.

## Highlights

• The incidence and prevalence of PBC in the Netherlands increased over the past decade.
• The age-dependent risk of PBC differed per sex; incidences were highest among females aged 45-64 years and males aged ≥65 years.
• PBC prevalence increased mainly in the elderly, in whom further evaluation of second-line treatment outcomes is needed.

## Graphical abstract

**Figure:**
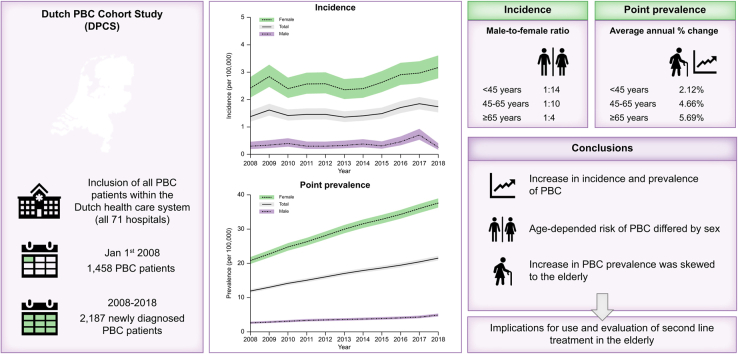

## Introduction

Primary biliary cholangitis (PBC) is a chronic cholestatic liver disease, with autoimmune features, characterized by progressive destruction of the intrahepatic bile ducts.^1^ PBC is predominantly diagnosed in middle-aged women and is notable for its insidious onset and slow progression, potentially leading to substantial liver-related morbidity and mortality.^2^ Understanding the incidence and prevalence of PBC is important for public health policies, clinical management strategies, and for implementing novel treatment options.

While PBC is considered a relatively rare disorder, accurate incidence and prevalence estimates remain challenging to obtain because of the limited comprehensive population-based registries. Recently, a systematic review and meta-analysis reported incidence and prevalence rates ranging from 0.23 to 5.31 per 100,000 and 1.91 to 40.2 per 100,000 inhabitants, respectively.^3^ There were marked geographical differences, with the highest incidence rates and prevalences in Northern America and Northern Europe. The lowest rates were previously reported in the Asia-Pacific region but were corrected more recently for China and Japan (prevalence 19.1 per 100,000 persons).^4^ Over the past few decades, the worldwide incidence and prevalence of PBC has been observed to increase in parallel with improvements in the methodology and quality of epidemiological studies. However, most epidemiological studies on PBC were performed in smaller geographic areas, so that the observed incidence rates and prevalence had to be translated to the national situation.^3^^,^^5^^,^^6^ In addition, the majority of these studies were performed in tertiary centers, potentially leading to referral and selection biases. Therefore, we aimed to assess the incidence and prevalence of PBC in the whole population of the Netherlands over time through the nationwide Dutch PBC Cohort Study (DPCS).

## Patients and methods

### Study design and study population

Patients were derived from the DPCS database. The DPCS is a nation-wide retrospective cohort study which includes every identifiable patient with an established diagnosis of PBC in the Netherlands with the first identifiable patient diagnosed in 1960. This study was conducted in all 71 hospitals in the Netherlands. Given the universal availability and reimbursement of healthcare services to all Dutch residents, private clinics for specialized hepatology care are practically non-existent within our health system. Diagnosis of PBC therefore typically occurs within the outpatient hospital setting under the care of gastroenterologists, hepatologists or internal medicine specialists, following referral of patients by the general practitioners (or other healthcare providers) for the evaluation of elevated liver enzymes. Patients with PBC who are diagnosed and fully managed outside of regular secondary care would not be included in the current study. However, this would be a rarity.

The study was conducted in accordance with the principles of the Declaration of Helsinki. The protocol was approved by the Medical Ethics Assessment Committee (METC) of the corresponding center and approved by the research board of each participating center, in accordance with their local regulations.

### Data collection

Between 2019 and 2022, comprehensive and systematic case identification was performed in all 71 hospitals in the Netherlands. Case identification was based on diagnosis and treatment codes (codes 707 and 954 are specific for PBC, PSC and AIH), antimitochondrial antibody test results, and locally available patient lists based on liver disease diagnosis or outcome. Medical records of the identified cases were reviewed by two dedicated medical doctors from the research team. All patients with an established PBC diagnosis according to the internationally accepted guidelines^2^^,^^7^ were included. Data collected for the current analyses included sex, date of birth, date of diagnosis, treatment center, date of death, date of liver transplantation (LT), and reason for loss to follow-up.

### Statistical analysis

Incidence and prevalence assessments were restricted to the time period between January 2008 and December 2018 (the year prior to the start of data collection). In general, around 2008 the electronic patient records were introduced, making almost all patient records readily accessible and ensuring uniform and reliable case identification through medical chart review. Restricting the analyses to the time-period after 2008 minimizes the likelihood that a methodological limitation with respect to earlier case identification is of major influence on the change in epidemiological estimates over time. The incidence rates and prevalence of PBC were expressed per 100,000 inhabitants (per year). Estimates of the yearly incidence rate were calculated as the number of new cases diagnosed during a year divided by the Dutch population at risk on the 1^st^ of January of the corresponding year, and were calculated for the whole population and per sex, age category or geographical region. The point prevalence was calculated as the total number of (alive) persons with a PBC diagnosis divided by the population at risk (on the 1^st^ of January of the corresponding year). To calculate the prevalence, a patient was no longer considered as a PBC case in the Netherlands following LT or when moving abroad. Patients who were lost to follow-up for other reasons were included in the analyses until they reached their expected sex- and birth year-specific life expectancy. The life expectancy and yearly size of the total Dutch population (≥20 years of age) were retrieved from Statistics Netherlands (*Centraal Bureau voor Statistiek*, Den Haag, the Netherlands; StatLine (cbs.nl)). In a more restrictive sensitivity analysis, all patients lost to follow-up were censored at the time of the last date they were known to be alive.

Poisson regression was used to assess the incidence rates and prevalence, and to compare them between the sex and age groups (20-44, 45-64, and ≥65 years) over time. In addition, (time) trends in incidence rates and point prevalences in a specific time period were estimated using Joinpoint regression by calculating the annual percent change (APC).^8^

For geographical analyses, the cohort was divided into four regions based on the location of the hospital where the patient was diagnosed with PBC. The regions (north, east, south, west) were pre-specified by Statistics Netherlands (Fig. S1). Differences in the median PBC incidence rates and point prevalences were calculated using the Kruskal-Wallis test.

All statistical tests were two-sided, and a *p* value <0.05 was considered statistically significant. For analyses with Joinpoint, the exact *p* value could not always be estimated by the program. In that case, *p* <0.05 was noted if the *p* value was statistically significant. Statistical analyses were performed using SPSS Statistics (version 28.0, IBM Corp.) and the Joinpoint Regression Program, Version 5.0.2, May 2023.

## Results

### Cohort characteristics

In total, 4,351 patients with a confirmed diagnosis of PBC were identified in all 71 hospitals in the Netherlands. Of these patients, 3,835 (88.1%) were female, and the mean age at PBC diagnosis was 57.1 (SD 12.7) years. On the 1^st^ of January 2008, there were 1,458 patients with PBC without LT in medical specialist care in 71 hospitals of the Dutch healthcare system. Between January 2008 and December 2018, a total of 2,187 patients were newly diagnosed with PBC, 307 (14.0%) were 20-45 years, 1,162 (53.1%) were 45-65 years, and 717 (32.8%) were ≥65 years old. Among these patients, 1,927 (88.1%) were female, and the mean age at diagnosis was 58.7 (SD 12.6) years. In the time period from 2008 to 2018, 46 (1.3%) patients underwent transplantation and 468 (13.7%) patients died, while 271 (7.9%) were lost to follow-up. Of the patients lost to follow-up, 73 (27.0%) went to the general practitioner, 18 (6.6%) moved abroad, and 33 (12.2%) were lost to follow-up for a variety of reasons. In addition, 147 (54.2%) patients were lost to follow-up for unknown reasons.

### Incidence of PBC in the Netherlands

**Fig. 1:**
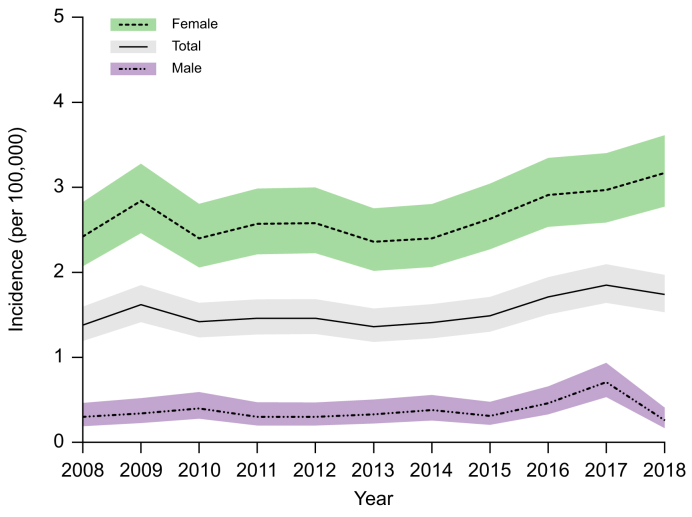
Incidence of primary biliary cholangitis stratified for sex. Yearly incidence rates with corresponding confidence intervals are presented per 100,000 inhabitants overall and according to sex (incidence rates were assessed using Poisson regression).

The incidence of PBC in 2008 was 1.38 per 100,000 inhabitants which increased to 1.74 per 100,000 inhabitants in 2018 (Fig. 1). The median yearly incidence rate was 1.46 (IQR 1.41-1.71) per 100,000 inhabitants. During the study period, the incidence of PBC increased with an age- and sex-adjusted annual incidence rate ratio (IRR) of 1.017 (95% 1.01-1.03, *p =* 0.014) (Table 1). However, Joinpoint modeling showed that the increase in PBC incidence was not stable over time as the APC was -0.63 (95% CI -10.35 to 3.58, *p* >0.05) from 2008 to 2014 and 6.86 (95% CI 1.50-18.79, *p* <0.05) from 2014 to 2018.

**Table 1:** Incidence and prevalence of primary biliary cholangitis per sex and age categories.

	Incidence per 100,000 in 2008	Incidence per 100,000 in 2018	Annual IRR∗	95% CI	*p* value
Total	1.38	1.74	1.017	1.01-1.03	0.014
Sex					
Female	2.42	3.17	1.015	1.00-1.03	0.035
Male	0.30	0.26	1.024	0.99-1.07	0.227
Age					
<45 years	0.56	0.42	0.986	0.95-1.02	0.451
45-64 years	2.12	2.81	1.023	1.00-1.04	0.015
≥65 years	1.91	2.29	1.019	0.10-1.04	0.110
	**Point prevalence per 100,000 in 2008**	**Point prevalence per 100,000 in 2018**	**AAPC**	**95% CI**	***p* value**
Total	11.93	21.53	5.94	5.77-6.15	<0.05
Sex					
Female	20.82	37.61	5.91	5.73-6.11	<0.05
Male	2.64	4.90	5.53	4.50-6.70	<0.05
Age					
<45 years	2.42	3.04	2.12	1.34-2.95	<0.05
45-64 years	16.36	25.91	4.66	4.47-4.94	<0.05
≥65 years	25.63	45.17	5.69	5.32-6.36	<0.05

Level of significance: *p* <0.05 (Poisson regression for IRR, Joinpoint regression for AAPC).

AAPC, average annual percent change; IRR, incidence rate ratio.

∗ Adjusted for age, sex and year (where applicable).

The yearly incidence of PBC was 2.42 per 100,000 female inhabitants *vs*. 0.30 per 100,000 male inhabitants in 2008, and 3.17 *vs.* 0.26 per 100,000 female and male inhabitants, respectively, in 2018 (Table 1). The age-adjusted annual IRR was 1.015 (95% CI 1.001-1.30, *p =* 0.035) for females and 1.024 (95% CI 0.99-1.07, *p* = 0.227) for males. The incidence estimates and annual adjusted IRR for each sex and age group are described in Table 1.

Subsequently, sex and age groups were compared using Poisson regression. The adjusted IRR of PBC for females was 6.98 (95% CI 6.14-7.95, *p* <0.001) as compared to males. Overall, the male-to-female ratio among the newly diagnosed patients with PBC was 1:7.4 during the study period. Stratified for age category, the adjusted IRR for females (*vs*. males) ranged from 14.51 (95% CI 9.22-22.83, *p* <0.001) in the population aged 20-44 years to 3.56 (95% CI 2.95-4.30, *p* <0.001) in the population aged ≥65 years (Table 2). In line with these results, the male-to-female ratio among newly diagnosed patients with PBC was 1:14.4 in patients aged <45 years, 1:9.9 in patients aged 45-64 years, and 1:4.4 in those aged ≥65 years.

**Table 2:** Primary biliary cholangitis incidence rate ratio and male-to-female ratio.

	IRR∗	95% CI	*p* value	Male-to-female ratio
Total				
Females *vs*. males	6.98	6.14-7.95	<0.001	1:7.4
<45 years				
Females *vs*. males	14.51	9.22-22.83	<0.001	1:14.4
45-64 years				
Females *vs*. males	9.94	8.15-12.13	<0.001	1:9.9
≥65 years				
Females *vs.* males	3.56	2.95-4.30	<0.001	1:4.4

Level of significance: *p* <0.05 (Poisson regression for IRR).

IRR, incidence rate ratio.

∗ IRR (time period 2008-2018) adjusted for age, sex and year (where applicable).

Overall, the adjusted IRR was 4.34 (95% CI 3.83-4.92) for the population aged 45-64 years and 4.11 (95% CI 3.60-4.70) for the population aged ≥65 years compared to the population aged <45 years, *p* <0.001 for both. Table 3 shows how the adjusted IRR for age categories differed according to sex. In females, the risk of a PBC diagnosis peaked in the 45-64 years age group (IRR 4.21, 95% CI 3.76-4.71, *p* <0.001 compared to those <45 years), with a decrease in risk among females ≥65 years (adjusted IRR compared to those aged 45-65 years: 0.83, 95% CI 0.75-0.92, *p* <0.001). In contrast, in males, the risk of a PBC diagnosis continued to increase with age. Compared to males <45 years, the IRR of a new PBC diagnosis was 6.30 (95% CI 4.18-9.51, *p* <0.001) for those aged 45-64 years and 14.41 (95% CI 9.62-21.60, *p* <0.001) for those ≥65 years.

**Table 3:** Primary biliary cholangitis incidence stratified for sex and age categories.

	Incidence 2008 per 100,000 inhabitants	Incidence 2018 per 100,000 inhabitants	Diagnosis 2008-2018 *(n)*	IRR§	95% CI
Female population					
<45 years	0.98	0.80	287	*reference*	
45-64 years	3.82	5.21	1,055	4.21∗	3.76-4.71
≥65 years	3.05	3.89	584	3.50∗	3.10-3.95
Male population					
<45 years	0.14	0.04	20	*reference*	
45-64 years	0.44	0.41	107	6.30∗	4.18-9.51
≥65 years	0.39	0.40	133	14.41∗	9.62-21.60

Level of significance: *p* <0.05 (Poisson regression for IRR).

IRR, incidence rate ratio.

§ IRR (time period 2008-2018) adjusted for age, sex and year (where applicable).

∗ *p* value <0.001 for all.

### Prevalence of PBC in the Netherlands

**Fig. 2:**
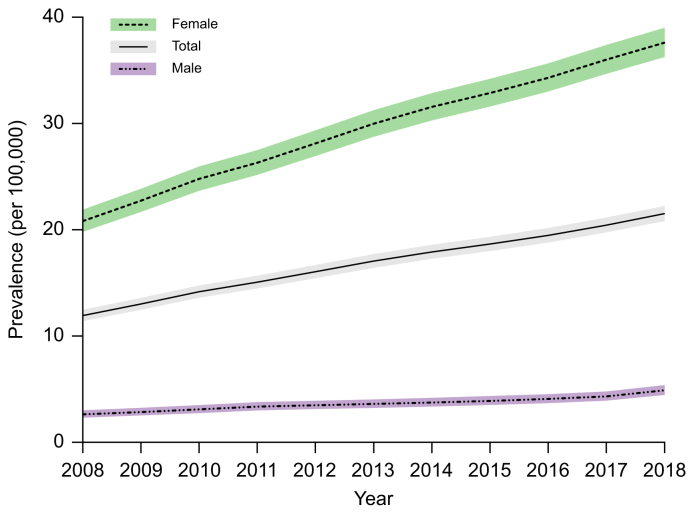
Prevalence of primary biliary cholangitis stratified for sex. The yearly point prevalence with corresponding confidence intervals are presented per 100,000 inhabitants overall and according to sex (point prevalence was assessed using Poisson regression).

The point prevalence of PBC in the Netherlands increased from 11.93 per 100,000 in 2008 to 21.53 per 100,000 inhabitants in 2018 (Fig. 2). The median yearly point prevalence was 17.06 (IQR 14.17-19.47) per 100,000 inhabitants. Joinpoint analysis showed an average APC from 2008 until 2018 of 5.94% (95% CI 5.77-6.15, *p* <0.05); this was 7.73% (95% CI 7.06-8.66, *p* <0.05) between 2008 and 2012 and 4.77% (95% CI 4.42-5.09, *p* <0.05) between 2012 and 2018. A more restrictive sensitivity analysis, in which patients lost to follow-up were censored at the date they were last known to be alive, showed similar results (Fig. S2).

The point prevalence was 20.82 and 37.61 per 100,000 female inhabitants, and 2.64 and 4.90 per 100,000 male inhabitants in 2008 and 2018, respectively. The male-to-female ratio among all patients with PBC in our nationwide cohort on the 1^st^ of January 2008 was 1:7.9. The average APC of the PBC prevalence showed a comparable increase in females (5.91, 95% CI 5.73-6.11, *p* <0.05) and males (5.53, 95% CI 4.50-6.70, *p* <0.05). Point prevalences of 2008 and 2018 and the average APCs are shown in Table 1.

When stratified by age, the point prevalence in 2018 ranged from 3.04 per 100,000 inhabitants aged <45 years to 45.17 per 100,000 inhabitants aged ≥65 years. Over time, compared to the population aged <45 years, the sex-adjusted prevalence ratios were 7.31 (95% CI 6.94-7.69) and 11.32 (95% CI 10.76-11.90) for those aged 45-64 years and ≥65 years, respectively. The increase in prevalence during the study period differed with age, with higher AAPC in older subgroups: 2.12% (95% CI 1.34-2.95, *p* <0.05) in those aged <45 years up to 5.69 (95% CI 5.32-6.36, *p* <0.05) in those ≥65 years (Table 1).

### Geographical differences in PBC epidemiology

The median incidence rate and prevalence per region are presented in Table 4. There were no statistically significant differences in the incidence rates or prevalences of PBC among the four regions (*p =* 0.286 and *p* = 0.712, respectively). The calculated median incidence rate and prevalence per region were all within the interquartile range of the national incidence rate and prevalence.

**Table 4:** Median incidence rates and prevalence per region.

	Incidence rate		Prevalence	
	Median	IQR	Median	IQR
North	1.43	1.20-1.80	15.84	13.19-18.55
East	1.62	1.37-1.92	17.45	14.53-20.63
South	1.68	1.42-1.83	16.56	13.49-19.78
West	1.48	1.35-1.57	17.38	14.54-19.02

Incidence rates and point prevalence in the time period of 2008-2018 were assessed using Poisson regression.

## Discussion

Based on this large, nationwide cohort study, we showed that there has been an increase in incidence and point prevalence of PBC in the Netherlands over the past decade. Because of our thorough and structural case finding, including medical chart review, in all hospitals within the healthcare system of a single country, our national estimates can be considered as highly reliable. In 2018, the yearly incidence was 1.74 per 100,000 inhabitants and the point prevalence was 21.53 per 100,000 inhabitants. In addition, we found that the age-dependent incidence rates differed according to sex. Within the female population, the highest PBC incidence was among those aged 45-64 years, whereas the incidence of PBC increased substantially with age in the male population, with the highest risk in males aged ≥65 years. As a result, the male-to-female ratio among newly diagnosed patients with PBC depended on age (ranging from 1:14.4 for those aged <45 years to 1:4.4 in the population aged ≥65 years). With respect to the point prevalence of PBC, the highest increase over the study period was found in the population aged ≥65 years. This might have implications for the use of second-line treatment options, and requires future studies to elaborate on the advantages and disadvantages in the elderly population.

Over ten years ago, Boonstra *et al.* showed an increasing prevalence of PBC in the Netherlands from 2000 to 2008. On the 1^st^ of January 2008, they estimated the PBC point prevalence in the Netherlands to be 13.2 per 100,000 inhabitants based on their data collection in a selection of 44 Dutch hospitals.^9^ Our national estimate of 11.9 per 100,000 in 2008 is only slightly lower, possibly because we also included all of the smallest centers. Since then, the prevalence has further increased to 21.5 per 100,000 inhabitants in January 2018, resulting in an increase of almost 6% per year in the past decade. Apart from the small increase in PBC incidence, this increased PBC prevalence may be explained by the improved survival of patients with PBC due to long-term use of adequate ursodeoxycholic acid treatment.^10^ In addition, prior studies have indicated that, after 2000, patients were increasingly diagnosed with earlier and less active disease.^11^ Both these factors are associated with beneficial clinical outcomes. As a result, these patients are more likely to remain in the Dutch healthcare system over time, thereby contributing to the increase in PBC prevalence. It was not unexpected that the increase in the number of patients with PBC was skewed toward the elderly population. However, this does have implications with respect to the use and evaluation of emerging second-line treatment options. The field is moving to more stringent biochemical goals,^12^^,^^13^ such as complete normalization of alkaline phosphatase (ALP). However, complete normalization of ALP may not be associated with substantial gain in survival as compared to ALP <1.5x the upper limit of normal among patients aged >62 years, which is in contrast to what was found in younger patients.^14^ More data on the biochemical and clinical efficacy of these drugs in the older PBC population are thus needed, as well as with respect to their side effects. Considering that our analyses were restricted to the years prior to 2018, it is unlikely that the use of second-line treatment options had a major influence on the observed increase in PBC prevalence. In the Netherlands, obeticholic acid is not available. Alternatively, off-label fibrate treatment can be considered. However, bezafibrate was only used sporadically during the timeframe of this study. This may be partially explained by the fact that a pivotal randomized-controlled clinical trial indicating the beneficial effect of bezafibrate on ALP (the most relevant surrogate marker in PBC) was published in 2018.^15^

Our results are also in line with those of a recent worldwide systematic review and meta-analysis on the epidemiology of PBC. This study showed an increasing incidence over the past decades with a pooled yearly incidence estimate in Europe of 1.86 per 100,000 persons (range 0.77-2.35).^3^ Our robust methodology, however, adds to the reliability of the current epidemiological estimates. The observed increase in PBC incidence reported by Lv *et al.* was mainly attributed to better disease awareness and improved search strategies. We cannot rule out that these factors have affected our results, even though we specifically focused on epidemiological changes after the introduction of electronic patient records in 2008. This has allowed for a stable and structured method for case finding. While the field of cholestatic liver diseases has indeed gained further attention due to drug development for second-line treatment in addition to UDCA in the last couple of years, viral hepatitis dominated hepatology up to 2018 (the last year of inclusion in our study). Furthermore, we included all patients with PBC in the smallest centers as well, which accounts for the majority of cases with a new diagnosis. Increased awareness is therefore unlikely to be the only explanation for the increase in the incidence of PBC over time, which was specifically found from 2014 onwards (APC of 6.68%).

The increase in the number of patients with PBC may also be related to a change in (the exposure to) environmental risk factors. In order to understand the pathophysiology of PBC, many studies have focused on potential triggers of PBC. Over the years, multiple potential factors have been described, such as smoking, recurrent urinary tract infections, use of exogenous estrogens, and chemicals (including hair dye and nail polish).[16], [17], [18], [19] Although their association with PBC development is still debated, changes in exposure to these factors over the last 20-30 years may contribute to the mild increase in PBC incidence in our cohort.^20^^,^^21^ This would also be true for PBC risk factors which have yet to be identified. In this respect, Dyson *et al.* showed that PBC was more prevalent in urban areas, especially in regions with a strong coal-mining heritage.^22^ In our study, the incidence and prevalence estimates did not differ according to the area in the Netherlands, but our analyses we were limited by the crude geographical analysis mapping available from Statistics Netherlands unlike the detailed analyses in the UK report.^22^

Notably, we observed a marked difference in the age-dependent PBC incidence between men and women. While the risk of PBC continues to increase with age in males, the risk of PBC peaks in middle-aged women. This could be explained by a difference in the timing of exposure to potential environmental triggers of PBC, although it may also be explained by delayed PBC diagnosis in male patients. A potential pathophysiological explanation concerns the sex-related differences in sex-binding hormone levels. Several studies have shown that women with PBC had lower testosterone levels than controls.^23^^,^^24^ Our finding of an increased risk of PBC in older men would be in line with the hypothesis that lower testosterone levels are related to susceptibility to PBC. In line with this, we found that the overall male-to-female ratio was 1:9. However, this ratio decreased with age; the ratio was 1:14 in those <45 years, 1:10 in those aged 45-64 years, and 1:4 in those ≥65 years.

Although the methodology and large size of this study are important strengths, there are some limitations that should be acknowledged. Our search for patients with PBC was thorough and comprehensive through various structural and predefined search strategies. A recent meta-analysis by Lv *et al.* showed that incidence and prevalence estimates were higher (and therefore more accurate) when multiple case finding methods, including those based on diagnostic treatment codes and anti-mitochondrial antibody test results, were used.^3^ While these were indeed the basis of our case finding strategy, we extended our search with locally available registrations based on liver disease diagnosis and/or outcome. Although, in our experience, this was the most feasible case finding methodology, we are unaware of its sensitivity to identify every single patient with PBC in each treatment center. It thus remains possible that we missed some patients with PBC, but we consider it unlikely that this would have a major impact on our results. All patients with PBC are indicated to be managed by a medical specialist, preferably a hepatologist or gastroenterologist. This is usually done within a secondary care setting and we included every hospital in the Netherlands in this this effort. Potential patients with PBC who did not enter the secondary medical care setting around diagnosis or during their follow-up are currently not included in the study. This clinical scenario is very unlikely for the population with PBC due to the structure of our healthcare system, which does not include private practices for specialized hepatology care. It is more likely that some patients with PBC in the Netherlands are yet to be diagnosed, although overcoming these limitations is practically impossible. This results in a slight underestimation of the true incidence rates and point prevalences. Further, due to the retrospective design of this study, some patients were lost to follow-up. It was decided to censor patients who were lost to follow-up in the year they reached their sex- and birth year-specific life expectancy. A sensitivity analysis, in which all patients lost to follow-up were censored at the date of their last visit, showed similar results, indicating the reliability of this approach. Also, due to legal restrictions, it was not possible to collect data on patients’ places of residence based on their postal codes. Therefore, in-depth analyses of geographical differences as potential environmental risk factors could not be performed in this study.

In conclusion, in this comprehensive nationwide cohort study, we observed an increase in both the incidence and prevalence of PBC in the Netherlands over the past decade. There were substantial differences in the age-dependent risk of PBC between females and males, with the highest incidence among the middle-aged population in females and among the elderly population in males. As expected, the overall growing number of patients with PBC was skewed toward the elderly population, which may have implications for the use and evaluation of second-line treatment.

## Abbreviations

AAPC, average annual percent change; ALP, alkaline phosphatase; APC, annual percent change; DPCS, Dutch PBC Cohort Study; IRR, incidence rate ratio; PBC, primary biliary cholangitis.

## Financial support

The current work within the Dutch PBC Cohort Study was partly funded by an unrestricted grant from Zambon Nederland B.V. in 2019 and 2020, and an unrestricted grant from CymaBay Therapeutics in 2021, 2022, and 2023.

## Conflict of interest

Adriaan J. van der Meer received grants from: CymaBay Therapeutics, Intercept Pharmaceuticals, Gilead Sciences, MSD and Zambon Nederland B.V; is consultant for Intercept Pharmaceuticals, Advanz Pharma, AOP Health, CymaBay Therapeutics, and Ipsen, and receives speakers fee from Zambon Nederland B.V. and AOP Health.

Ulrich Beuers reports grants from ZonMW Netherlands and Amsterdam UMC Foundation and receives consulting fees from Abacus and Behring and speakers fee from GSK and Zambon. Cyriel Y. Ponsioen received research grants from Perspectum and Gilead consultancy fees from Chemomab and NGM, and speakers fee from Tillotts. Harry L.A. Janssen received grants from: Gilead Sciences, GlaxoSmithKline, Janssen, Roche, Vir Biotechnology Inc and is consultant for: Aligos, Gilead Sciences, GlaxoSmithKline, Grifols, Roche, Vir Biotechnology Inc., Precision Biosciences. All other authors report no potential conflict of interest for this manuscript.

Please refer to the accompanying ICMJE disclosure forms for further details.

## Authors’ contributions

Study concept and design: RdV, MH, EW, AM, UB, JD, FC, BH, BV, MK, SM, RV, HF, JV, TG, CP. Data acquisition: all authors. Data Analysis: RdV, EW, MH, AM. Data Interpretation: RdV, EW, MH, AM. Drafting manuscript RdV, AM, EW, MH. Critical revision for important intellectual content and final approval: all authors.

## Data availability statement

As this is a multicenter study in which data among 71 centers is shared, the dataset which was generated is not openly available for researchers outside of the study team. Please contact the corresponding author to inquire about the possibilities of collaboration.

## Collaborators

All collaborating authors of the Dutch PBC Study Group.

Sunje Abraham; Rob P.R. Adang; Huseyin Aktas; Yasser A. Alderlieste; Maria N. Aparicio-Pages; L. (Bert) C. Baak; Martine A.M.C. Baven-Pronk; A. (Sander) van der Beek; Frank C. Bekkering; Jeroen D. van Bergeijk; Ulrich Beuers; Menno Beukema; Wink de Boer; Femke Boersma; Kirsten Boonstra; Frank ter Borg; Martijn J. ter Borg; Pieter C.J. ter Borg; Gijs J. de Bruin; Paul J. Bus; Djuna L. Cahen; Marcel Cazemier; Frans J.C. Cuperus; Lisette J.H. van Dam; Maaike J. Denters; Joost P.H. Drenth; Ludger S.M. Epping; Nicole S. Erler; Hajo J. Flink; Philip W. Friederich; Nicole F.M. van Gerven; Tom J.G. Gevers; Bettina E. Hansen; Sven J. van den Hazel; Bart van Hoek; Maria C. van Hooff; Daphne M. Hotho; Harry L.A. Janssen; Hendrik J.M. de Jonge; Matthias C. Jurgens; J.(Netty) van Kemenade; Marjo J. Kerbert-Dreteler; Michael Klemt-Kropp; Ingrid C.A.W Konings; Sander de Kort; Edith M.M. Kuiper; Johan P.H. Kuyvenhoven; Adriaan J. van der Meer; Suzanne van Meer; Susanne L. Onderwater; Leendert H. Oterdoom; Cyriel Y. Ponsioen; Paul G. van Putten; Janne E. van Rooij; Robert Roomer; Johannes Schmidt-Böhmer; Stephan Schmittgens; Tim C.M.A. Schreuder; Jerome Sint Nicolaas; Hanneke van Soest; Khalida Soufidi; Stephan H.C. van Stiphout; Hans H.K. Thio; Merel M. Tielemans; Sigrid Vandebosch; Rozanne C. de Veer; Bart J. Veldt; Robert C. Verdonk; J. Marleen de Vree; Elsemieke de Vries; Anne Vrieze; Jan Maarten Vrolijk; Laurens A. van der Waaij; Ellen Werner; Ulrike de Wit; Frank H.J. Wolfhagen.

## References

[1] Kaplan M.M., Gershwin M.E. (2005). Primary biliary cirrhosis. N Engl J Med.

[2] European Association for the Study of the Liver (2017). Electronic address eee, European Association for the Study of the L. EASL Clinical Practice Guidelines: the diagnosis and management of patients with primary biliary cholangitis. J Hepatol.

[3] Lv T., Chen S., Li M. (2021). Regional variation and temporal trend of primary biliary cholangitis epidemiology: a systematic review and meta-analysis. J Gastroenterol Hepatol.

[4] Zeng N., Duan W., Chen S. (2019). Epidemiology and clinical course of primary biliary cholangitis in the Asia-Pacific region: a systematic review and meta-analysis. Hepatol Int.

[5] Terziroli Beretta-Piccoli B., Stirnimann G., Cerny A. (2018). Geoepidemiology of primary biliary cholangitis: lessons from Switzerland. Clin Rev Allergy Immunol.

[6] Marzioni M., Bassanelli C., Ripellino C. (2019). Epidemiology of primary biliary cholangitis in Italy: evidence from a real-world database. Dig Liver Dis.

[7] Lindor K.D., Bowlus C.L., Boyer J. (2019). Primary biliary cholangitis: 2018 practice guidance from the American association for the study of liver diseases. Hepatology.

[8] Kim H.J., Fay M.P., Feuer E.J. (2000). Permutation tests for joinpoint regression with applications to cancer rates. Stat Med.

[9] Boonstra K., Kunst A.E., Stadhouders P.H. (2014). Rising incidence and prevalence of primary biliary cirrhosis: a large population-based study. Liver Int.

[10] Harms M.H., van Buuren H.R., Corpechot C. (2019 Aug). Ursodeoxycholic acid therapy and liver transplant-free survival in patients with primary biliary cholangitis. J Hepatol.

[11] Murillo Perez C.F., Goet J.C., Lammers W.J. (2018). Milder disease stage in patients with primary biliary cholangitis over a 44-year period: a changing natural history. Hepatology.

[12] Murillo Perez C.F., Harms M.H., Lindor K.D. (2020). Goals of treatment for improved survival in primary biliary cholangitis: treatment target should Be bilirubin within the normal range and normalization of alkaline phosphatase. Am J Gastroenterol.

[13] de Veer R.C., Harms M.H., Corpechot C. (2022). Liver transplant-free survival according to alkaline phosphatase and GLOBE score in patients with primary biliary cholangitis treated with ursodeoxycholic acid. Aliment Pharmacol Ther.

[14] Corpechot C., Lemoinne S., Soret P.A. (2024). Adequate versus deep response to ursodeoxycholic acid in primary biliary cholangitis: to what extent and under what conditions is normal alkaline phosphatase level associated with complication-free survival gain?. Hepatology.

[15] Corpechot C., Chazouilleres O., Rousseau A. (2018). A placebo-controlled trial of bezafibrate in primary biliary cholangitis. N Engl J Med.

[16] Corpechot C., Chretien Y., Chazouilleres O. (2010). Demographic, lifestyle, medical and familial factors associated with primary biliary cirrhosis. J Hepatol.

[17] Gershwin M.E., Selmi C., Worman H.J. (2005). Risk factors and comorbidities in primary biliary cirrhosis: a controlled interview-based study of 1032 patients. Hepatology.

[18] Parikh-Patel A., Gold E.B., Worman H. (2001). Risk factors for primary biliary cirrhosis in a cohort of patients from the United States. Hepatology.

[19] Matsumoto K., Ohfuji S., Abe M. (2022). Environmental factors, medical and family history, and comorbidities associated with primary biliary cholangitis in Japan: a multicenter case-control study. J Gastroenterol.

[20] Hair dye - Fast growing business (May 26, 2001). The Economist.

[21] Stefanick M.L. (2005). Estrogens and progestins: background and history, trends in use, and guidelines and regimens approved by the US Food and Drug Administration. Am J Med.

[22] Dyson J.K., Blain A., Foster Shirley M.D. (2021). Geo-epidemiology and environmental co-variate mapping of primary biliary cholangitis and primary sclerosing cholangitis. JHEP Rep.

[23] Kempinska-Podhorodecka A., Abramczyk J., Cielica E. (2023). Effect of low testosterone levels on the expression of proliferator-activated receptor alpha in female patients with primary biliary cholangitis. Cells.

[24] Floreani A., Paternoster D., Mega A. (2002). Sex hormone profile and endometrial cancer risk in primary biliary cirrhosis: a case-control study. Eur J Obstet Gynecol Reprod Biol.

