# Supplementary material for: Incidence and prevalence of primary biliary cholangitis in the Netherlands – A nationwide cohort study
Source: JHEP Rep. 2024 Jun 6;6(8):101132. doi: 10.1016/j.jhepr.2024.101132 (PMC11304051; doi:10.1016/j.jhepr.2024.101132)

Incidence and prevalence of primary biliary cholangitis in the Netherlands – A nationwide cohort study

Rozanne C. de Veer, Maria C.B. van Hooff, Ellen Werner, Ulrich Beuers, Joost P.H. Drenth, Frans J.C. Cuperus, Bart van Hoek, Bart J. Veldt, Michael Klemt-Kropp, Suzanne van Meer , Robert C. Verdonk, Hajo J. Flink, Jan Maarten Vrolijk, Tom J.G. Gevers, Cyriel Y. Ponsioen, Martijn J. ter Borg, Khalida Soufidi, Femke Boersma, Hendrik J.M. de Jonge, Frank H.J. Wolfhagen, L.C. Baak, Susanne L. Onderwater, Jeroen D. van Bergeijk, Paul G. van Putten, Gijs J. de Bruin, Rob P.R. Adang, Maria N. Aparicio-Pages, Wink de Boer, Frank ter Borg, Hanneke van Soest, Harry L.A. Janssen, Bettina E. Hansen, Nicole S. Erler, Adriaan J. van der Meer on behalf of the Dutch PBC Study Group

Table of contents

Fig. S1 2

Fig. S2. 2

Fig. S1. The Netherlands divided into four regions.

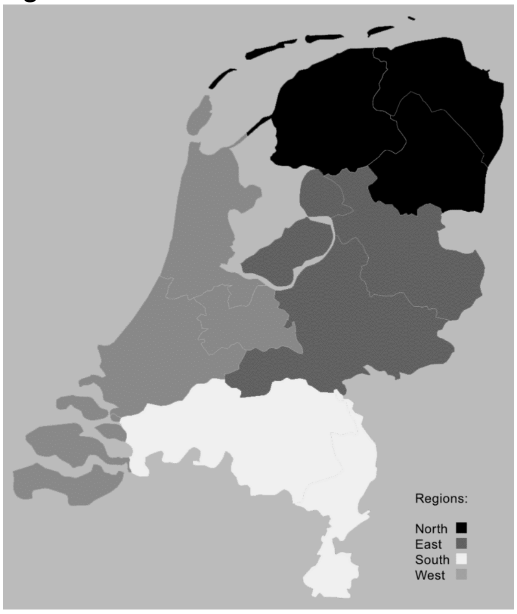

Fig. S2: Prevalence of PBC in the Dutch population.
The yearly point prevalence with corresponding confidence intervals are presented per 100.000 inhabitants overall and in the restrictive analysis (Poisson regression).

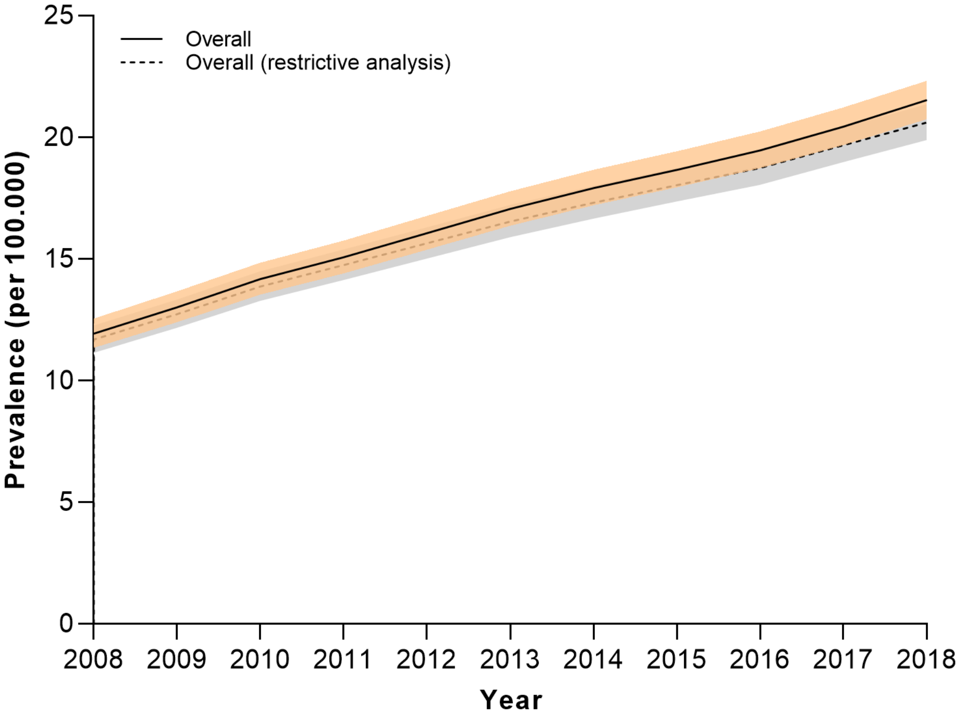

Supplement: Multimedia component 1 [file mmc1.pdf]
